# Synthetic electroretinogram signal generation using a conditional generative adversarial network

**DOI:** 10.1007/s10633-025-10019-0

**Published:** 2025-04-16

**Authors:** Mikhail Kulyabin, Aleksei Zhdanov, Irene O. Lee, David H. Skuse, Dorothy A. Thompson, Andreas Maier, Paul A. Constable

**Affiliations:** 1 Pattern Recognition Lab, Department of Computer Science, Friedrich-Alexander-Universität Erlangen-Nürnberg, Erlangen, Germany; 2 VisioMed.AI, Moscow, Russia; 3 Behavioural and Brain Sciences Unit, Population Policy and Practice Programme, UCL Great Ormond Street Institute of Child Health, University College London, London, UK; 4 The Tony Kriss Visual Electrophysiology Unit, Clinical and Academic, Department of Ophthalmology, Great Ormond Street Hospital for Children NHS Trust, London, UK; 5 UCL Great Ormond Street Institute of Child Health, University College London, London, UK; 6 College of Nursing and Health Sciences, Caring Futures Institute, Flinders University, Adelaide, 5000 Australia

**Keywords:** Neurodevelopment, Retina, Neural network, Biomarker, Waveform

## Abstract

**Purpose:**

The electroretinogram (ERG) records the functional response of the retina. In some neurological conditions, the ERG waveform may be altered and could support biomarker discovery. In heterogeneous or rare populations, where either large data sets or the availability of data may be a challenge, synthetic signals with Artificial Intelligence (AI) may help to mitigate against these factors to support classification models.

**Methods:**

This approach was tested using a publicly available dataset of real ERGs, *n* = 560 (ASD) and *n* = 498 (Control) recorded at 9 different flash strengths from n = 18 ASD (mean age 12.2 ± 2.7 years) and *n* = 31 Controls (mean age 11.8 ± 3.3 years) that were augmented with synthetic waveforms, generated through a Conditional Generative Adversarial Network. Two deep learning models were used to classify the groups using either the real only or combined real and synthetic ERGs. One was a Time Series Transformer (with waveforms in their original form) and the second was a Visual Transformer model utilizing images of the wavelets derived from a Continuous Wavelet Transform of the ERGs. Model performance at classifying the groups was evaluated with Balanced Accuracy (BA) as the main outcome measure.

**Results:**

The BA improved from 0.756 to 0.879 when synthetic ERGs were included across all recordings for the training of the Time Series Transformer. This model also achieved the best performance with a BA of 0.89 using real and synthetic waveforms from a single flash strength of 0.95 log cd s m^−2^.

**Conclusions:**

The improved performance of the deep learning models with synthetic waveforms supports the application of AI to improve group classification with ERG recordings.

## Introduction

The full field electroretinogram (ERG) is the electrical response of the retina recorded under dark- or light-adapted conditions in response to a brief flash of light [2]. The clinical ERG is typically used to support the diagnosis of an inherited or acquired retinal disease using time-domain parameters based on the main a- and b-wave peak amplitudes and times [3]. Differences in the ERG waveform has been proposed as a potential biomarker in a range of psychiatric conditions such as schizophrenia -reduced b-wave [4], depression [5], Parkinson’s Disease [6, 7], autism spectrum disorder (ASD) [8, 9] and attention deficit/hyperactivity disorder (ADHD) [9–11]. For reviews see [12–15]. In the case of ASD, the light-adapted b-wave amplitude and oscillatory potentials are reported to be reduced in this study population [8, 9] but not in a separate adult population [16]. Therefore, developing models that helps to classify and identify these conditions using the ERG may help to improve health outcomes in ASD [17, 18] and related neurological disorders [12–15].

The field of synthetic signal generation with Artificial Intelligence (AI) has received significant attention in recent years, prompting numerous advances in medical imaging [19] and retinal structural analyses [20]. Larger datasets developed with AI could improve the sensitivity and specificity of deep learning models by providing a larger and balanced training dataset using the combined real and synthetic ERG waveforms. For example, in the field of cardiology synthetic electrocardiogram (ECG) traces have been generated using a bidirectional long short-term memory (LSTM) and convolutional neural network (CNN) to improve models for disease classification [21]. Similarly, a one-dimensional auxiliary classifier Generative Adversarial Network (GAN) increased the sample of under-represented classes with synthetic ECG traces to improve classification in class-imbalanced populations [22]. Within the field of neurology, a variational auto-encoder with GAN has been applied to develop fake brain networks derived from functional MRI datasets to improve ADHD classification [23].

The introduction of synthetic ERGs is new to the field of visual electrophysiology and raises questions about their authenticity and potential clinical utility. The derivation of synthetic signals relies upon having a natural or real dataset initially from which to generate the synthetic waveforms. The use of synthetic ERG signals may support the augmentation of datasets to balance groups such as gender as previously shown [24]. They may also be used as in this case to support data intensive classification models using Deep Learning. There are likely to be other potential uses for synthetic ERGs developed in the future such as augmentation of clinical reference groups for comparison with disease. We appreciate that some skepticism may exist, and clinicians may view these synthetic waveforms as not a true physiological signal and therefore invalid. This demonstration of the use of synthetic ERGs aims to mitigate against some hesitancy and provides some evidence for their use.

This is the first work, to our knowledge, to apply a synthetic ERG generation method using AI to classify clinical groups. The main aim of this study was to assess the feasibility of synthetically generating ERG waveforms using a GAN to provide a larger reference dataset and to test this using open-source data in a neurodevelopmental condition. This report describes firstly: The introduction of a GAN to produce synthetic ERG waveforms corresponding to both ASD and control classes and secondly: The application of Transformer models using time–frequency and time domain features to classify the groups (classes) using real only or the real and synthetic ERG waveforms.

## Methods

### Participants

The ASD participants met DSM-IV or DSM-5 (Diagnostic and Statistical Manual of Mental Disorders) criteria based on assessment with ADOS or ADOS-2 (Autism Diagnostic Observation Schedule) and the developmental, dimensional and diagnostic interview (3Di, [25] assessed by pediatric psychiatrists or clinical psychologists based at the Great Ormond Street Hospital in the UK or local Child and Adolescent Mental Health clinics in Adelaide. The ASD group (n = 18) had an age (mean ± SD) 12.2 ± 2.7 with range 8.3–16.6 years with 9 male and 9 female participants. The typically developing Control group (n = 31) were recruited through social media and existing site databases and excluded those with any direct family history of ASD. The Control, group’s age was 11.8 ± 3.3 and ranged from 6.0–16.8 years with 23 male and 8 female participants. There was no significant difference between groups for age p = 0.69 (One way ANOVA) or sex assigned at birth p = 0.09 (Chi-Squared).

### Dataset

The open access raw ERG waveform dataset [1] is available at: https://doi.org/10.25451/flinders.27116719.v1.

ERGs were recorded under light adapted conditions from the right then left eye using a randomized series of nine white flash strengths (1.204, 1.114, 0.949, 0.799, 0.602, 0.398, 0.114, -0.119, -0.367 log cd.s.m^−2^ on a 40 cd.m^−2^ white background. The flashes were presented at 2 Hz using a custom Troland protocol with skin electrodes placed 2–3 mm below the lower lid with the RETeval (LKC Technologies, Gaithersburg, MD, USA) handheld unit. ERG waveforms averaged 30–60 traces per eye to generate the reported averaged waveforms that were used in the analysis. Waveforms with artifacts such as blinks were automatically rejected if they fell within the upper or lower quartile of the overall average. Replicate recordings within an eye, when recorded, were included in the total dataset for analysis. All signals had a length of 235, covering the time interval -20 ms to 100 ms, with the flash stimulus presented at t = 0. For further details see [8, 10, 26]. Table 1 summarizes the distribution of the number of real recorded ERG waveforms (Signals) for each flash strength for the ASD and Control groups used for synthetic signal generation.

**Table 1:** The number (*n*) of real electroretinogram waveform signals obtained from the ASD and Control groups for each of the nine flash strengths

Strength (log cd s m^−2^)	ASD (*n*)	Control (*n*)
1.204	67	57
1.114	65	54
0.949	60	55
0.799	63	55
0.602	64	58
0.398	65	55
0.114	53	50
− 0.119	56	50
− 0.367	52	53
Total (*n*)	560	498

### Deep learning

**Fig. 1:**
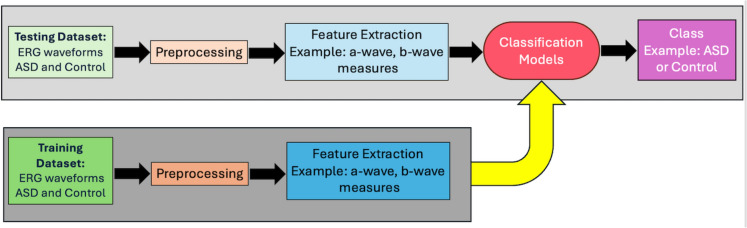
Schematic flow diagram of the traditional pattern recognition pipeline, that consists of the training and testing phases. The ERG waveforms are pre-processed to extract features of the waveform that can be used for training the classification models. These features can be selected based on their importance such as the b-wave amplitude or other time-domain properties extracted during the pre-processing of the waveform. These features are used to train the models to obtain the best classification performance (in this case identifying ASD from Control groups). A separate dataset is then used to ‘test’ the classification model using the same features to determine if the model can obtain accurate classification using the test data that is excluded from the training dataset that was used to train the classification models

Pattern recognition is a field of machine learning that focuses on identifying patterns or regularities in data and using these patterns to classify or make decisions about new, unseen data. Before the rise of deep learning, traditional pattern recognition relied on manually designed algorithms and models to extract meaningful features from raw data, which were then used by classifiers to make predictions, for example, separating pictures of cats from pictures of dogs. Traditionally, there are two phases in a pattern recognition pipeline: testing and training, as shown in Fig. 1. For a general introduction to deep learning methodology see Maier et al. (2019) [27].

The training dataset was first preprocessed during the training phase, and significant features were extracted. Preprocessing occurs within the original data and includes noise reduction and image correction tasks to improve data quality. Feature extraction involves identifying a set of distinctive and comprehensive characteristics that represent the data. For instance, in distinguishing between cats and dogs, features might include the shape of the nose. This process, often referred to as “handcrafting”, is highly specific to the application and usually requires designing new features for each case. Based on the extracted features, the classifier predicts the corresponding category by applying a function that produces the classification result. The classifier’s parameters are optimized during training and later tested on an independent dataset to evaluate performance. Deep learning revolutionized pattern recognition by automating feature extraction through neural networks. It allowed models to learn features and classifications simultaneously from raw data, thereby eliminating the need for manual feature engineering.

Deep learning has transformed how we approach various complex problems, making it possible to analyse, predict, and create in previously unimaginable ways. In recent years, deep learning has been used increasingly in the medical domain. One of the major areas is classification, where the goal is to sort data into categories, such as images of healthy and unhealthy scans [28–31].

### Deep Learning approach

The first step was to train the conditional generator adversarial network (CGAN) to produce ASD or control signals. Then, to prove that the synthetic signals had the same features as the real signals, we trained two neural networks (TST and ViT) to classify signals into the ASD and control classes. The neural networks trained on the dataset augmented with synthetic signals showed higher classification metrics, indicating the generated signals' quality. The test data were initially separated from the training dataset in both cases. If this separation into test and train subsets was not performed in advance then features of the test signals may appear in the synthetic signals, simplifying the task of the classification model and falsely overestimating the model's accuracy. This separation into test and training sets is a common approach when using generative models, such as with electrocardiogram signals to boost classification of disease [32].

**Fig. 2:**
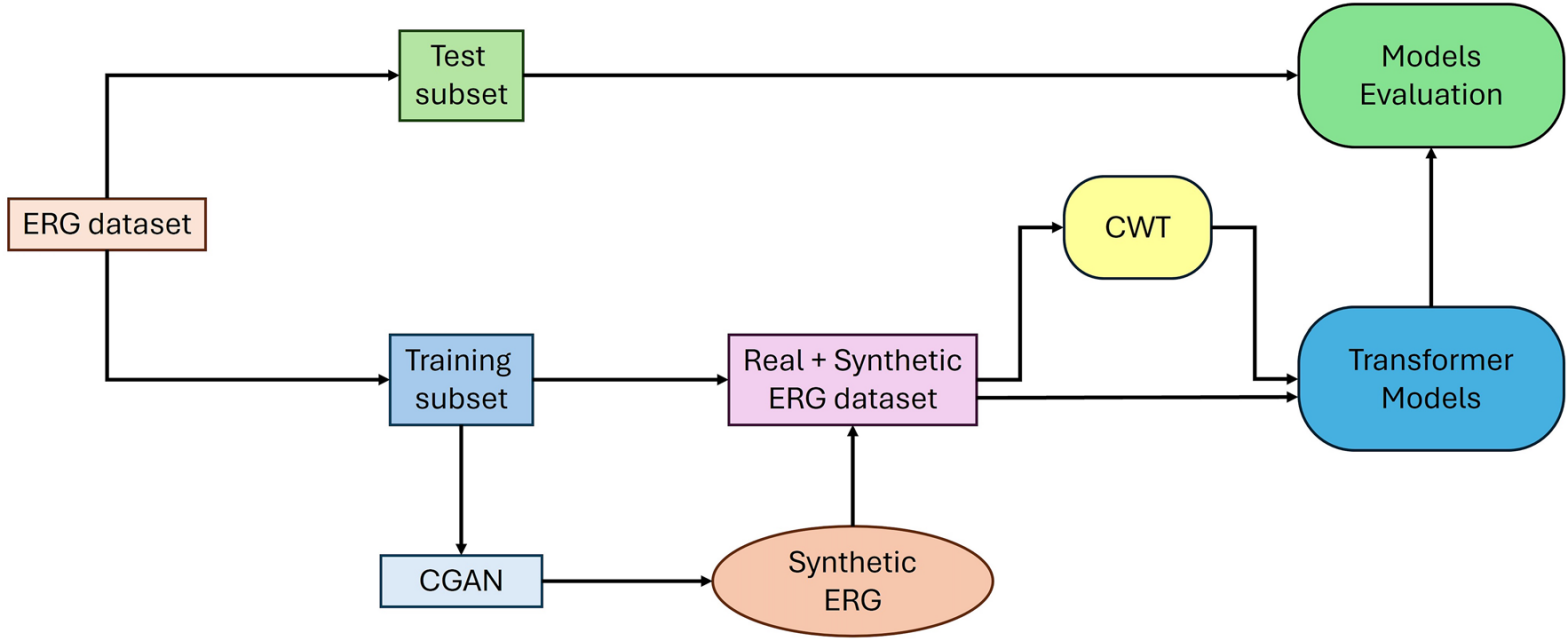
Method overview. The dataset was split into test and training subsets before Conditional Generative Adversarial Network (CGAN) training so that the generative model was not trained on data correlated to the test data for a fair evaluation. Synthetic signals generated by the CGAN model were added to real data. The classification models were then trained on the composite (combination of real and synthetic) and on the real only dataset. Two Transformer models were trained for classification and evaluation with obtained dataset: Visual Transformer (ViT) and Time Series Transformer (TST). ViT was trained on the time-frequency features obtained with Continuous Wavelet Transform (CWT), TST was trained directly on the time-series data

Consequently, 25% of the ERG waveform dataset from each group were partitioned as the test dataset and stored untouched for the future unbiased evaluation outcomes of the group classification. The remaining waveforms were utilized to train the CGAN to synthesize ERG signals. These synthetic signals were then combined with real signals to form an augmented dataset for training the Transformer-based classification models. For the training of the Visual Transformer (ViT) model, a Continuous Wavelet Transform (CWT) was applied, generating a wavelet representation for each signal. For the Time Series Transformer (TST) training, the ERG waveforms were used in their original time-series representation. The evaluation process used a five-fold cross-validation step, and the performance metrics were averaged over the folds. Figure 2 provides a schematic of the ERG waveform data handling.

## Conditional generative adversarial network (CGAN)

The framework for the synthetic waveform generation used a Conditional GAN (CGAN) to obtain the synthetic signals from the real ERG waveforms. The CGAN architecture comprised two sub-networks: The Generator (G) and the Discriminator (D). The goal of the Generator was to learn the transformation between the latent distribution *p*_*z*_ and the real-world data distribution *p*_*d*_. The Discriminator then learnt to distinguish real signals from the synthesized ones. The Generator and the Discriminator were provided with auxiliary class information (*y*) as an additional input layer with each class or group designated 0 or 1. The complete CGAN architecture was trained in a *min–max* optimization game as in the standard GAN loss function: the Discriminator tried to maximize the score for real signals, D(*x/y*) and minimize the score for the generated D(*G(z/y*)) signals. The Generator tried to minimize *log* (1 − D(G(z/y))), such that the minimizing of the term was only possible if the Generator synthesized realistic synthetic signals. The only difference with standard GAN was that the Conditional Probability was used for both the Generator and Discriminator instead of a regular one. The complete optimization process is shown in Eq. 1.

1 $$\begin{aligned}\frac{minmaxV\left(D,G\right)}{G D}&={E}_{x {p}_{d\left(x\right)}}\lceil logD\left(x/y\right)\rceil \\ &\quad+{E}_{z {p}_{z\left(z\right)}}\lfloor log\left(1-D\left(G\left(z/y\right)\right)\right)\rfloor \end{aligned}$$

The conditional vector is the main difference between the GAN and CGAN. Conditional vector is an additional input for both Generator and Discriminator models, so they already know which signal (ASD or control) they generate or discriminate. Consequently, the Generator learns to generate ASD and control signals as well as possible to defeat the Discriminator, which discriminates between the fake and real signals within both ASD and control classes. Ultimately, it is known which signal has been generated, and so the ground truth is known a priori.

## Recurrent neural network (RNN)

**Fig. 3:**
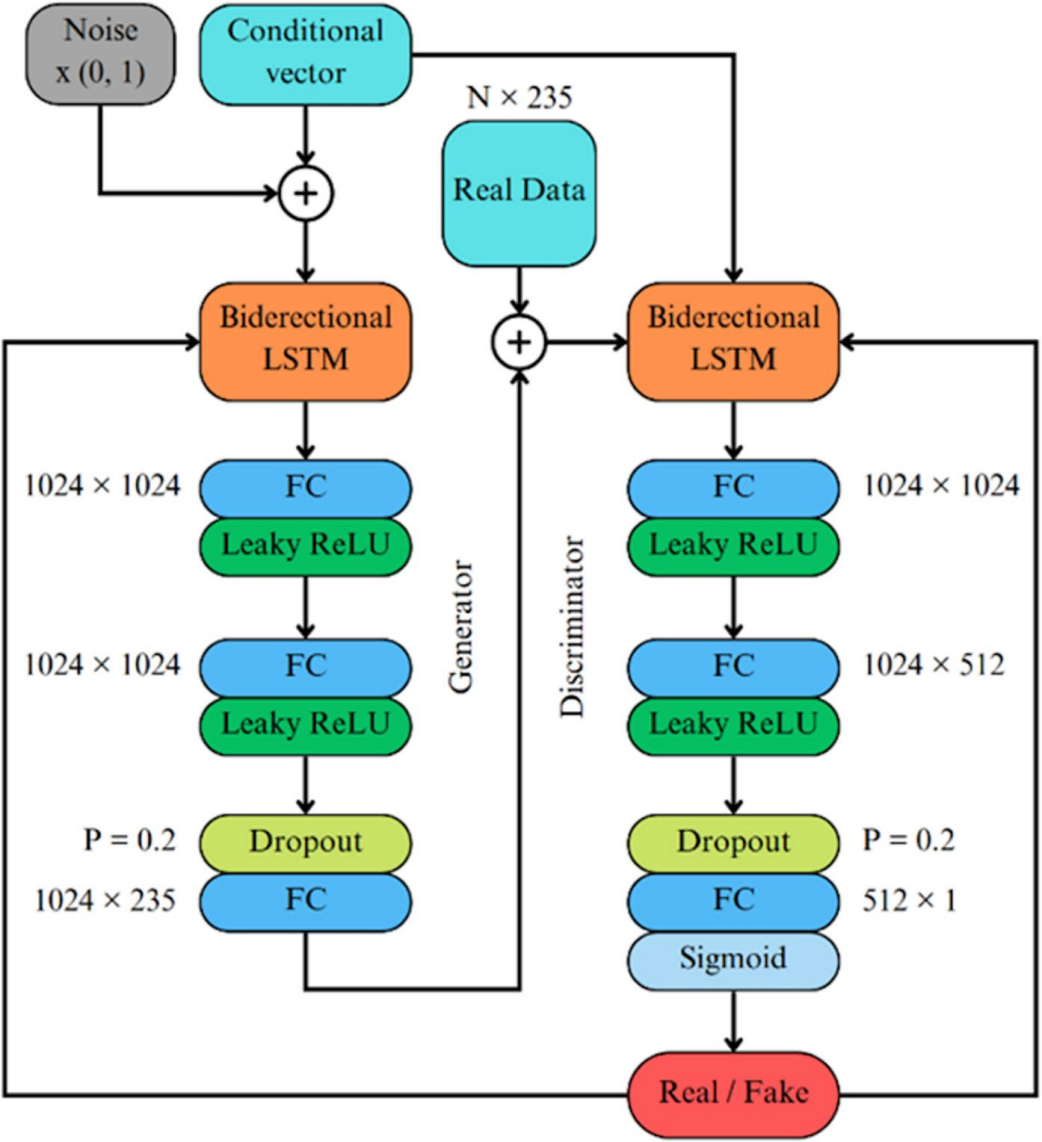
The structure of the Conditional Generative Adversarial network (CGAN) for generation of synthetic electroretinogram waveforms. CGAN consists of Generator (left) and Discriminator (right) modules. Both modules consisted of the combinations of BLSTM and fully connected (FC) layers. Conditioning was achieved by adding a conditional vector

Recurrent Neural Networks (RNNs) have gained widespread adoption in diverse applications, including time series data processing, speech recognition, and image generation [33]. While RNNs exhibit proficiency in handling short-term dependencies, they have limitations in effectively addressing long- term dependencies. To overcome these limitations, Long-Short Term Memory (LSTM) networks were introduced as an extension of RNNs [34]. LSTM incorporates a memory cell architecture that retains prior contextual information, thus accommodating issues such as gradient expansion or disappearance during training [35]. A Bidirectional LSTM (BLSTM) is a neural network technique that facilitates bidirectional sequence information flow, encompassing both backward and forward directions [36]. In contrast to the conventional LSTM architecture, where the input is unidirectional, either flowing backward or forward, BLSTM allows for concurrent information propagation in both directions. This bidirectional flow ensures the preservation and utilization of future and past contextual cues within the network, which distinguishes BLSTM from its unidirectional LSTM counterpart and was incorporated into the CGAN structure. Figure 3 depicts the CGAN structure used that consisted of a BLSTM layer of size 235 × 512, with two blocks of fully connected (FC) layers of size 1024 × 1024, and a Leaky Rectified Linear Unit (ReLU) activation function. The dropout was then followed by another FC layer of size 1024 × 235. The Discriminator had the same structure except for the sizes of the FC layers (1024 × 1024, 1024 × 512 and 512 × 1). An additional Sigmoid function was used to scale the output to the range of 0 to 1.

**Fig. 4:**
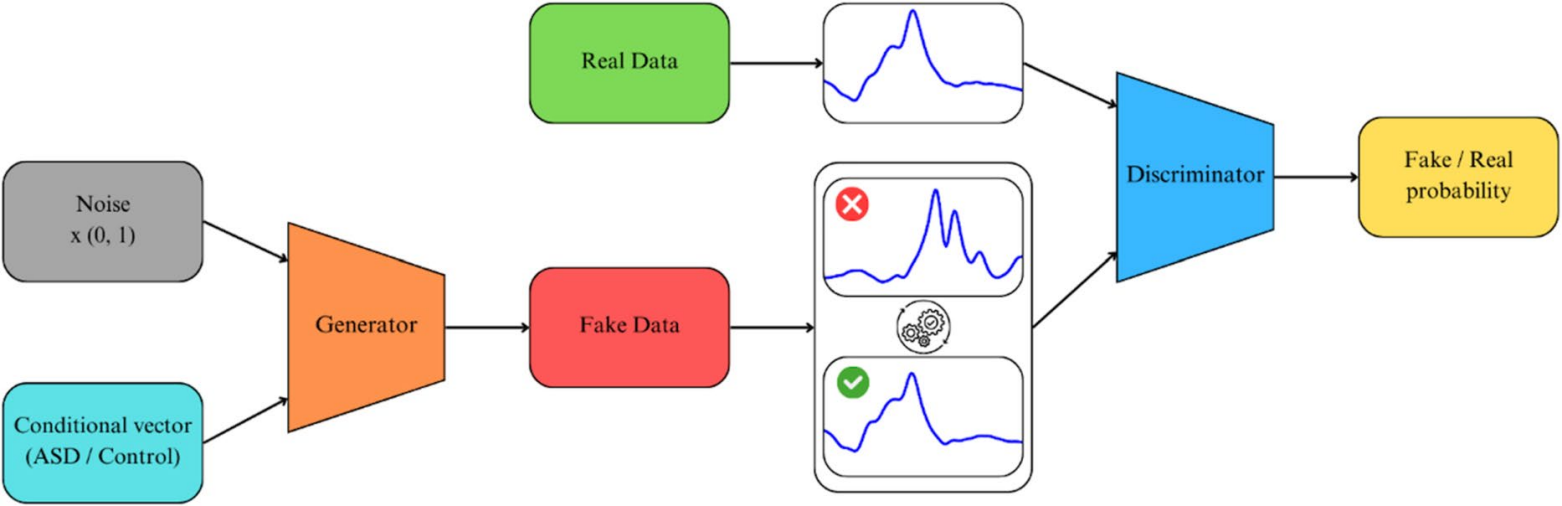
An overview of the CGAN optimization process. CGAN consists of two modules: Generator and Discriminator. Generator synthesizes fake ‘synthetic’ signals based on noise and condition inputs, while the Discriminator evaluates both natural and synthetic signals to determine authenticity, refining the Generator’s ability to create realistic ERG signals over time

Figure 4 shows the overall scheme of the CGAN generation process. Generator takes random noise and a condition vector (ASD/Control) to synthesize fake data resembling the real ERG signals. Simultaneously, the Discriminator distinguishes between natural and synthetic signals, outputting the data's probability of being real. Both networks are trained in opposition: the generator improves its ability to create realistic data, while the discriminator becomes better at detecting fake data. Over time, this optimization process allows the generator to create highly realistic signals that the discriminator struggles to identify as fake. Then, it is possible to obtain synthetic ERG signals from noise by specifying only the signal's membership in a particular group.

### Synthetic waveforms

**Fig. 5:**
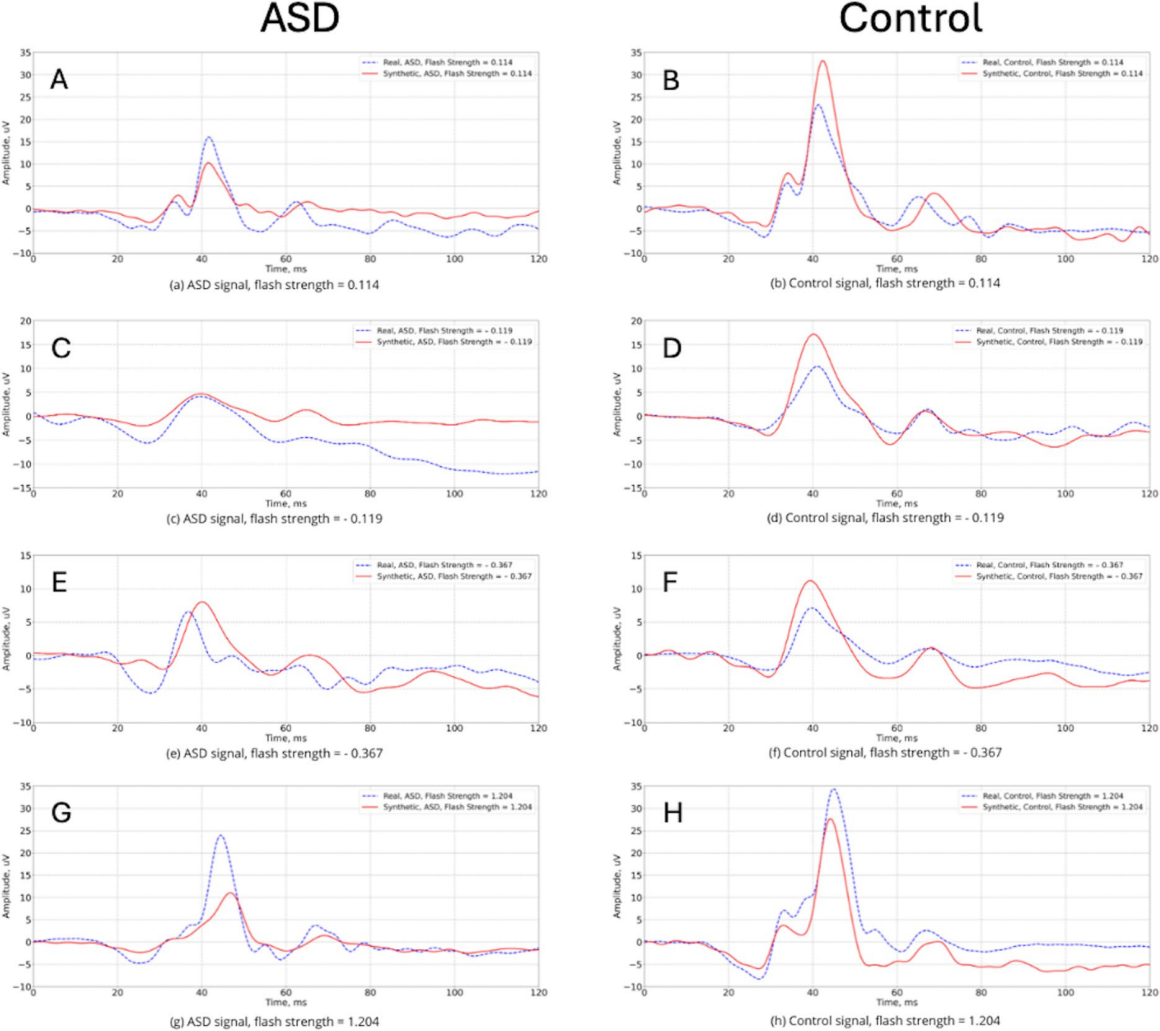
Representative examples of the real and synthetically generated by CGAN ERG signals for ASD and Control groups respectively at flash strengths 0.114 (**a** and **b**),—0.119 (**c** and **d**),—0.367 (**e** and **f**) and 1.204, (G and H) in log cd s m^−2^. The solid red lines represent synthetic ERG signals, dashed blue lines are real signals. Note baseline is 0 to 20 ms preceding the stimulus flash

Using the model, synthetic ERG waveforms were produced for each class of the ASD and Control groups. Examples of the generated synthetic and real signal waveforms are shown in Fig. 5. The solid-colored lines represent synthetic ERG waveforms, and the dashed black lines the real ERG waveforms. The synthetically generated ERG waveforms exhibited the key morphological features of the real ERGs recorded in the participants. Notably, the high-frequency component of the oscillatory potentials on the ascending limb of the b-wave was present. Additionally, the a- and b-wave amplitudes were in close agreement with the natural ERG signals, although the time to peaks were more variable.

The size of the initial dataset limits the possible number of generated signals. As mentioned above, we initially split the dataset into the training and test subsets with a ratio of 75:25. Consequently, we have 743 signals in the train and 248 for the test. For further training of the classificational model, we used the ratio of real to synthetic/generated signals of ~ 2:1 with 370 generated signals in total correspondingly. The limitation is not the ratio between the real and synthetic signals for the training, but the size of the training dataset will generate a larger range of synthetic signals to capture the variance in the population.

### Continuous wavelet transform (CWT)

To decompose the raw time series signal *x(t)* into the time–frequency domain a CWT of the ERG waveform signal was applied to provide an overcomplete representation of the signal by letting the translation and scale parameters of the wavelets vary continuously. The CWT of the function *x(t)* at a scale (a > 0) $\in$ R^+∗^ with the translational value b $\in$ R was expressed by the integral, where *ψ(t)* is the continuous function called the mother wavelet, and the overlying tilde (ῶ) represents the operation of the complex conjugate [37]. The output of the CWT consisted of a two- dimensional time-scale representation of the signal defined by Eq. 2. The primary objective of the mother wavelet was to serve as the foundational function for generating daughter wavelets, which are simply the translated and scaled versions of the mother wavelet.

2 $${X}_{w} \left(a,b\right)= \frac{1}{{|a|}^{1/2}}{\int }_{-\infty }^{\infty }x(t){ \tilde{\omega } } \left(\frac{t-b}{a}\right)dt$$

Using the method previously described [38], the three best mother functions for the dataset were the Ricker, Gaussian, and Morlet based on the obtained classification accuracies. To increase the efficiency of the input wavelet image for further classification the input consisted of three wavelets as three channels [39, 40].

### Visual transformer (ViT)

**Fig. 6:**
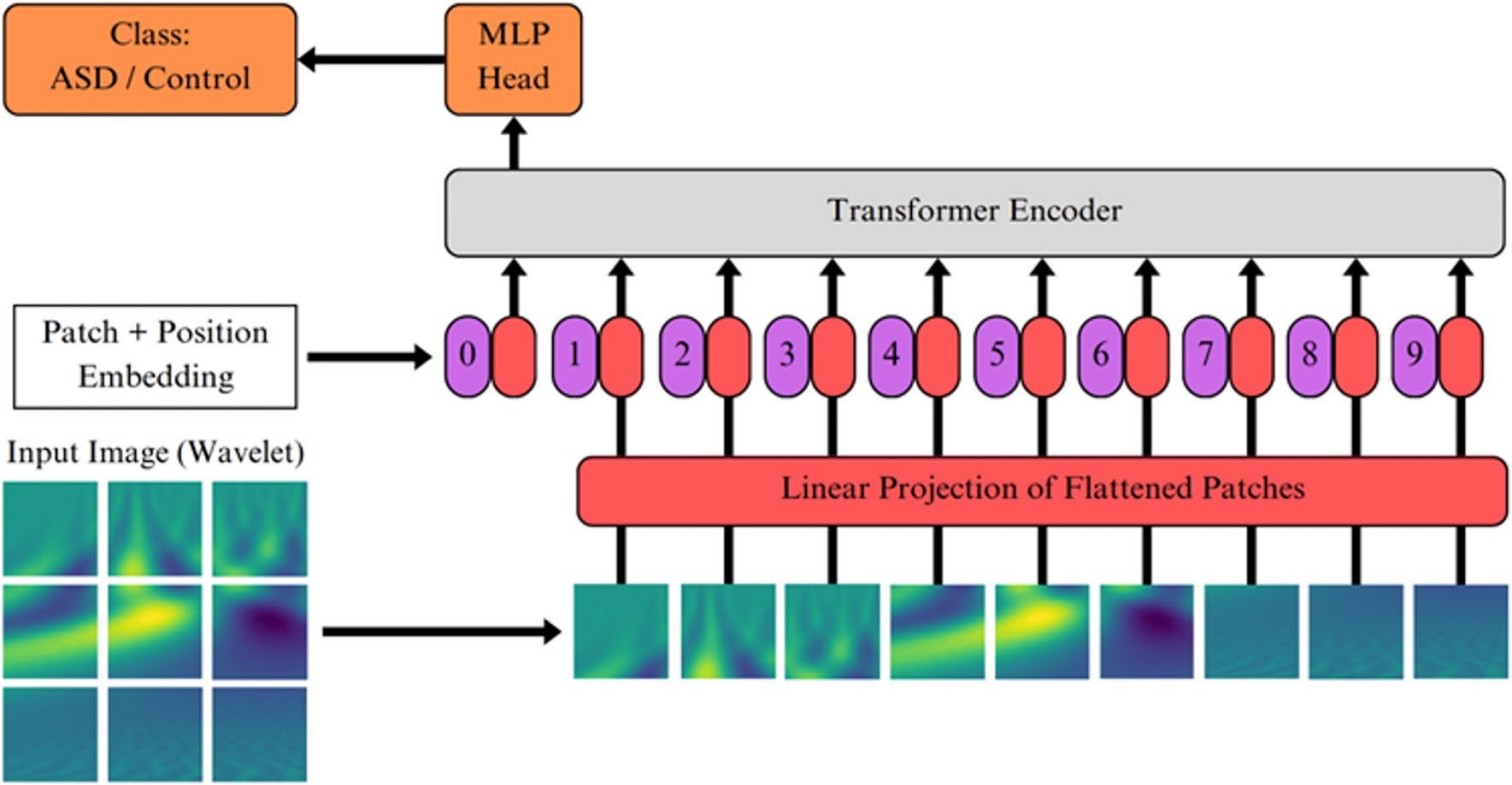
The Visual Transformer with 2D patches from signal analysis using CWT of the electroretinogram waveforms. 2D image (wavelet) is initially transformed into a sequence of the paths which were then mapped into a constant latent vector. This vector is an input of Transformer encoder, which consisted of alternating layers of a multiheaded self-attention and a multilayer perceptron block, that outputs a class of the wavelet using a Multi-Layer Perceptron (MLP) head

Transformers have emerged as the preferred model for image classification tasks, primarily attributed to their computational efficiency and scalability [41, 42]. The ViT was applied to wavelet scalograms using real and synthetic datasets. Figure 6 illustrates the ViT architecture proposed by Dosovitskiy et al. (2020) [43]. Initially, the model processed a two-dimensional (2D) input image (wavelet in this case) by transforming it into sequences of flattened 2D patches. These patches underwent a trainable linear projection to map them into a constant latent vector before processing the patches through the encoder, where a learnable embedding was added to the beginning of the sequence to retain positional information. The sequence of embedding vectors served as inputs to the Transformer encoder, which consisted of alternating layers of a multiheaded self-attention and a multilayer perceptron block [43]. For the classification task, the image representation was passed through a classification head for fine-tuning.

The input sequence can be formed from feature maps of a CNN instead of raw image patches by leveraging a convolutional backbone [44]. In this case, the patch embedding projection was applied to patches extracted from a CNN feature map. This hybrid approach of a Residual Network (ResNet) and ViT enhanced the model’s capability to handle smaller-sized images and improved the overall parameter efficiency.

### Time series transformer (TST)

TST is a neural network architecture designed to process and analyze time series data and is based on the Transformer architecture introduced by Vaswani et al. (2017) [42] for natural language processing tasks, but without the decoder part of the architecture [45]. TST adapts the Transformer architecture to handle sequences of temporal data points effectively, such as those found in time series biological signals such as the ERG. As with the original Transformer model, TST uses self-attention mechanisms to weigh the importance of different elements within the input time series sequence. This attention mechanism allows the model to learn dependencies between different time steps and capture long-range dependencies. Positional encodings are crucial to TST and are added to the input data to provide further information about the sequence’s order of the time steps. The TST model also employed a multi-head attention mechanism to simultaneously capture different relationships and representations within the ERG time series data.

### Model training

The CGAN was trained with a batch size of 15 on 10,000 epochs with a dropout of 0.2. An Adam optimizer was used with a learning rate of 0.0002 for the Generator and the Discriminator, with the Binary Cross Entropy Loss (BCELoss) used as the criterion function. The training time of the CGAN model was two hours using an AMD Ryzen 95900HX × 16 processor with an NVIDIA GeForce RTX 3070 graphics card. The model had 10^6^ trainable parameters with a complexity of 0.026 Gigaflops.

For the classification, two (Small and Tiny), ResNet-ViT hybrid image models were used which differed in their number of parameters and have previously shown effectiveness in ECG wavelet classification [32]. The two models were ViT S (ViT_small_r26_s32_224) and ViT T (ViT_tiny_r_s16_p8_224), available at the HuggingFace ‘transformers’ repository [45]. Both models were pretrained on ImageNet-21 k and fine-tuned with ImageNet-1 k with additional augmentation and regularization with a resolution of 224 × 224 pixels. A Stochastic Gradient Descent (SGD) optimization with a 0.001 initial learning rate was used. ViT S and ViT T had 36.4 × 106 and 10.4 × 106 trainable parameters with complexities of 3.5 and 0.4 Gigaflops, respectively. For TST, a cross-entropy loss function with class weights adapted to address the class imbalance and an Adam optimizer with an initial learning rate of 0.0001 was used. Each model was then trained until convergence using the early stopping criteria on the validation loss with a batch size of 32.

The models were evaluated using a five-fold cross-validation. Performance metrics were Balanced Accuracy (BA), Precision (P), Recall (R), F1-score, and Area Under the Curve (AUC) as defined in Eqs. 3–8, where TP = True Positive, TN = True Negative, and FP = False Positive and FN = False Negative.

3 $$Precision=\frac{TP}{TP+FP}$$

4 $$Recall=\frac{TP}{TP+FN}$$

5 $$F1Score=\frac{2xPrecisionxRecall}{Precision+Recall}$$

6 $$Balanced\,Accuracy\left(BA\right)=\frac{Sensitivity+Specificity}{2}$$

where:

7 $$Sensitivity=\frac{TP}{TP+FN}$$

8 $$Specificity=\frac{TN}{TN+TP}$$

The performance outcomes were averaged across the five-folds for different test conditions for the real only and the combined real and synthetic ERG waveforms. The TST was trained for each strength independently, as well as on all strengths simultaneously. ViT was trained only on the entire signal dataset (all 9 flash strengths). The input for the ViT model was the wavelet scalograms obtained using CWT, and the input for TST was the ERG signals in their time series representation. BA was used as the main outcome measure.

## Results

### Model performance

The best BA achieved for the real only data set for each model was 0.805 (TST) at single strength of 1.114 log cd.s.m^−2^ and 0.777 for Vit-Small using all nine flash strengths. In contrast, when the combined real and synthetic data sets were used then the BA improved for both models. In the case of the TST the BA increased to 0.891 for the 1.114 log cd.s.m^−2^ (15% higher). The best BA of 0.894 was obtained with the single strength (0.949 log cd.s.m^−2^) using the TST model with combined real and synthetic waveforms. In the case of the ViT models, the BA also increased to 0.883 (14% increase) when all strengths were used with the combined real and synthetic ERG waveforms. In comparison the TST model, when all flash strengths were included from the real only dataset did not perform as well with a BA of 0.756. See Table 2 for full details of the network performance metrics.

**Table 2:** Summary performance results of the Time Series Transformer (TST) and Visual Transformer (ViT) network classification models using the real only or real and synthetic electroretinogram datasets

Network	Strength	Real only					Real + synthetic				
		BA	P	R	F1	AUC	BA	P	R	F1	AUC
TST	1.204	0.710	0.710	0.736	0.717	0.739	0.842	0.933	0.736	0.823	0.897
TST	1.114	***0.805***	0.789	0.833	0.810	0.768	***0.891***	0.900	0.947	0.923	0.916
TST	0.949	0.759	0.727	0.888	0.799	0.845	***0.894***	0.857	0.947	0.914	0.969
TST	0.799	0.722	0.700	0.777	0.736	0.753	0.815	0.772	0.894	0.829	0.850
TST	0.602	0.722	0.750	0.666	0.705	0.842	0.842	0.842	0.842	0.842	0.950
TST	0.398	0.712	0.710	0.737	0.731	0.725	0.815	0.815	0.731	0.774	0.955
TST	0.114	0.749	0.723	0.731	0.749	0.744	0.763	0.812	0.784	0.742	0.817
TST	− 0.119	0.750	0.736	0.777	0.756	0.867	0.815	0.833	0.789	0.810	0.955
TST	− 0.367	0.722	0.666	0.888	0.761	0.756	0.879	0.861	0.842	0.900	0.953
TST	All	***0.756***	0.716	0.849	0.777	0.852	***0.879***	0.874	0.859	0.876	0.948
ViT S	All	***0.777***	0.761	0.759	0.759	0.836	***0.883***	0.895	0.873	0.882	0.958
ViT T	All	0.757	0.759	0.757	0.757	0.814	0.873	0.873	0.873	0.873	0.945

*AUC* area under the curve, *BA* Balanced Accuracy, *F1* F1 score, *P* Precision, *R* Recall, *Strength* Flash strength in log cd s m^−2^

### Synthetic waveforms

**Fig. 7:**
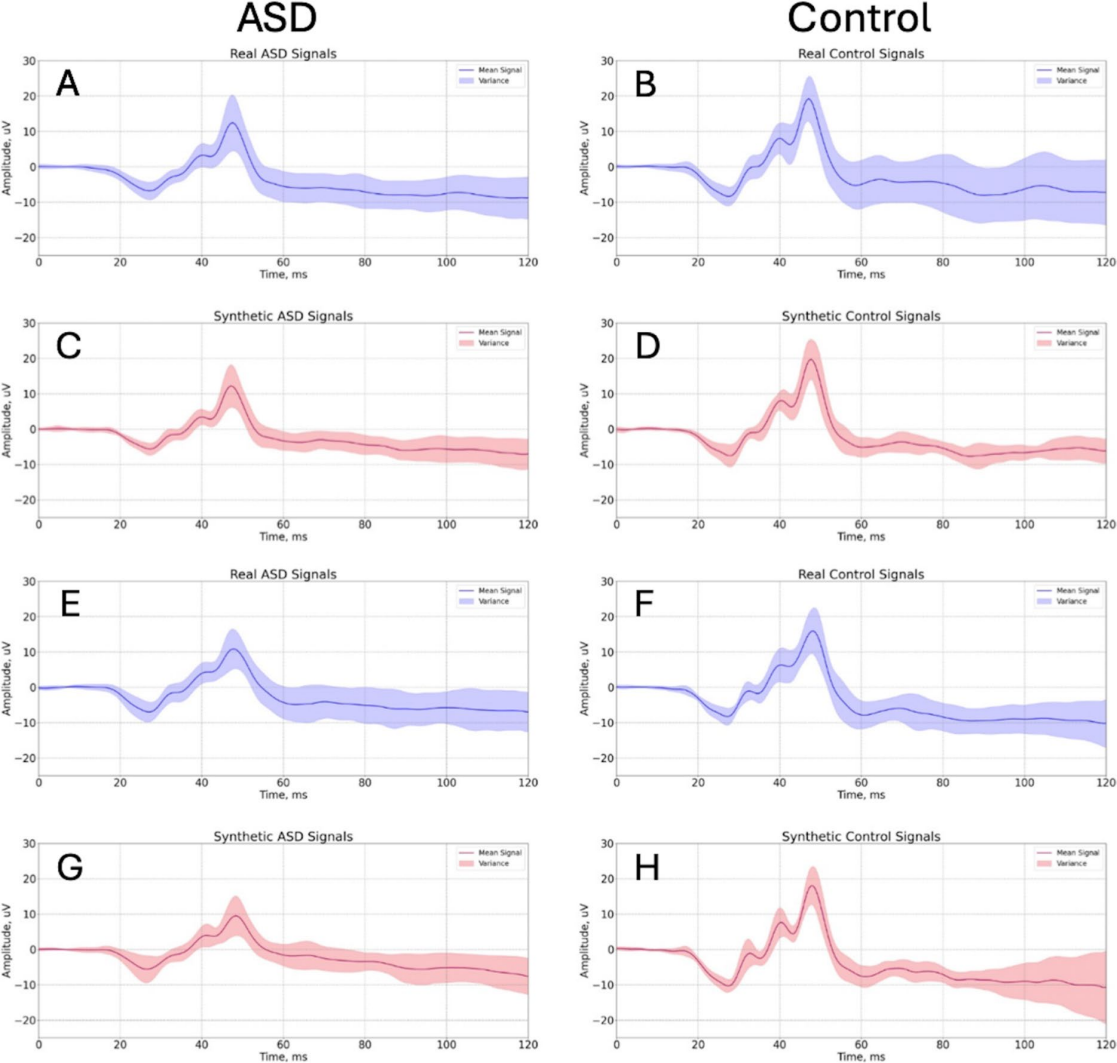
Mean and variance plots of the Real (blue) and synthetic (pink) ERGs for the Control group at 1.114 (**a**–**d**) and the 1.204 (**e**–**h**) log cd s m^−2^ flash strengths. The synthetic ERG waveforms were generated using CGAN and filtered with a Butterworth low-pass filter at 300 Hz cutoff frequency

Figure 7 shows the synthetic and real ERG examples from the Control group generated using CGAN and then filtered using a 300 Hz Butterworth low-pass filter for the 1.114 and 1.204 log cd.s.m^−2^ strengths. Although morphologically similar there was typically greater variance in the synthetic signals after 60 ms. The Synthetic dataset of 1.204, 1.114, 0.949, and 0.799 (log cd.s.m^−2^) flash strengths are available on DataPort [1] for both the ASD and Control groups.

**Fig. 8:**
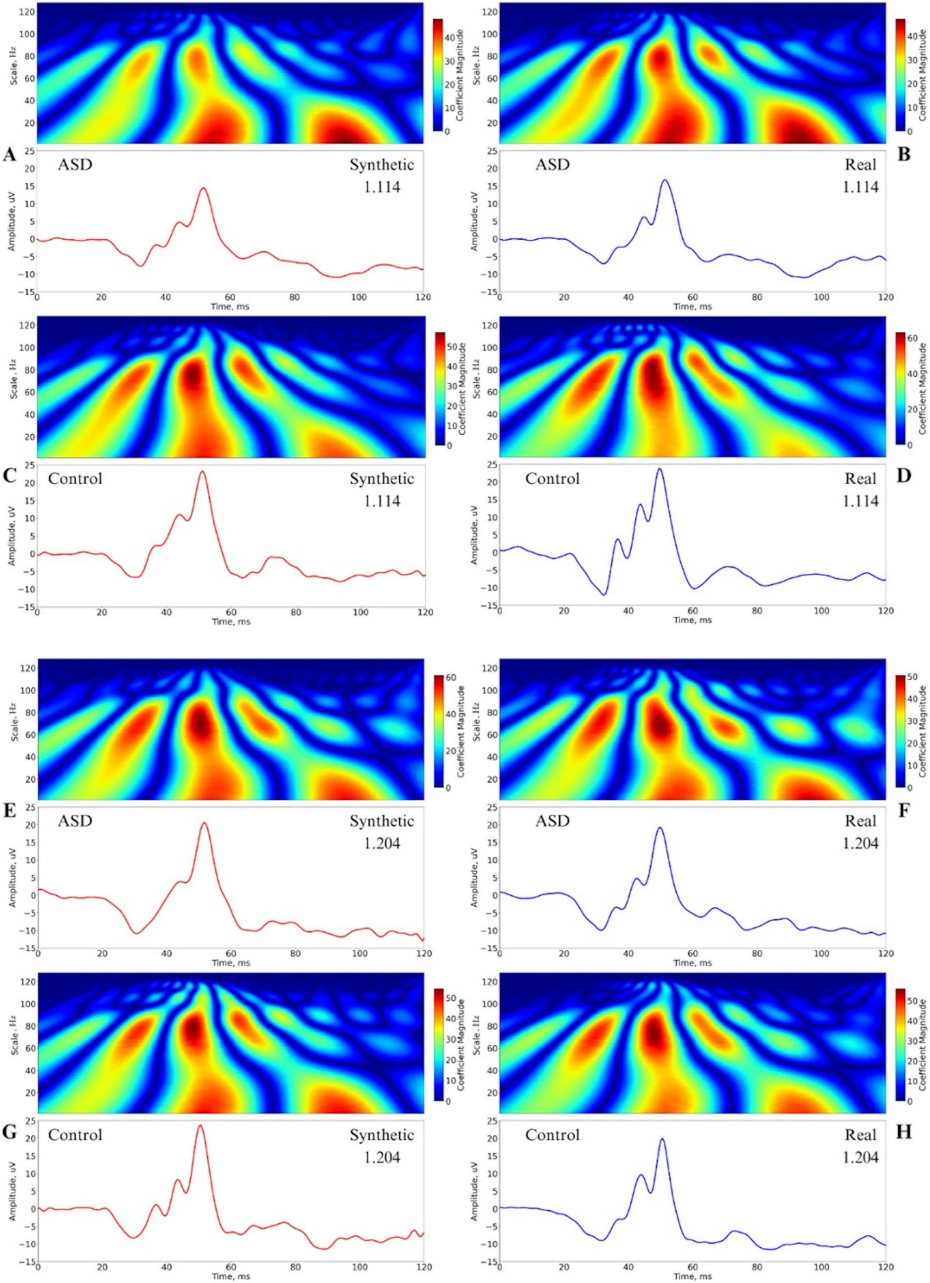
Decomposition of real and synthetic waveforms at 1.114 (**a**–**d**) and 1.204 (**e**–**h**) log cd s m⁻^2^ flash strengths from representative ERG waveforms of ASD and Control groups. Time–frequency spectral scalograms were generated using the Continuous Wavelet Transform (CWT) with a Morlet mother wavelet. The Y-axis represents the wavelet scale, inversely related to frequency, facilitating analysis of how different frequency components evolve. The magnitude of the wavelet coefficients indicates the strength of these components at specific times and scales

Figure 8 shows the CWT decomposition with the Morlet mother wavelet for a representative waveform that is real and synthetic for the flash strengths of 1.114 and 1.204 log cd.s.m ^2^ from the ASD and Control Group. The Y-axis denotes the wavelet scale, which inversely correlates with frequency. The relationship between scale (*a*) and frequency (*f*) is given by Eq. 9, where *fc* is the center frequency of the Morlet wavelet function. In this analysis, the signal length is 120 samples, and the sampling period is calculated accordingly.

9 $$f=\frac{{f}_{c}}{a\bullet [sampling period]}$$

The scales range from 1 to 128. The coefficient magnitude represents the strength of the correlation between the wavelet at a specific scale and position with the signal, indicating the presence and intensity of a particular frequency components at specific times. This representation allows for the analysis of how different frequency components of the signal evolve. A more formal analysis of the spectral and time domain features will be conducted in the future to compare the characteristics of the real and synthetic waveforms. The pattern of the decomposed signals was similar between the real and synthetic signals.

## Discussion

The integration of synthetic ERG signals with real signals can boost the overall classification accuracy for groups. In all cases explored in this analysis, the BA was greater when the real and synthetic waveforms were used with an improvement in the BA by up to 14%. In comparison to previous analyses, using data derived from the same clinical population, with two flash strengths the best BA was 0.82 [46]. In a separate analysis using a gated multilayer perceptron network analysis achieved a BA of 0.89 but required all nine flash strengths [47]. In this case the same BA was achievable with time series data from a single flash strength at 0.949 log cd.s.m^−2^ with the combined real and synthetic waveforms. Thus, augmenting real with synthetic data can reduce the need for multiple recordings at different flash strengths to obtain the same classification accuracy as previously reported [47].

This is the first work to propose a synthetic ERG generation method using AI for the classification of ASD using a functional response of the retina. Given the quest for an early diagnostic biomarker for ASD then the use of AI generated synthetic signals may enhance the sensitivity and specificity of biomarkers for ASD and potentially related neurological disorders [14, 15]. Further applications may be to balance datasets for factors that affect the ERG amplitude such as age [48, 49], birth sex [50], iris color [51], pupil diameter [52] dark adaptation interval [53], skin electrode placement [54] and the type of electrode [55]. Thus, CGAN offers the possibility of generating data sets specific to these variables to mitigate against testing conditions or population characteristics. This may be of relevance to ASD and ADHD where there is a male sex bias for diagnosis [56] and balancing the comparison group for sex assigned at birth may not always be possible. The incorporation of AI generated synthetic ERG waveform generation could be used to augment and balance clinical groups [24] to increase the performance of classification models as shown in this case.

The identification of a clinically viable biomarker for ASD is lacking despite multiple studies investigating molecular and genetic [57] markers and biological signals including eye tracking, fMRI or EEG signals [58] as potential biological markers [59]. However, no ideal biomarker has been identified that has been translated into clinical practice. The ERG findings add to the growing list of potential biomarkers for ASD and there is some early evidence that the ERG may also help in the classification of ADHD [10, 11, 60]. This new approach to handling limited-sized datasets that utilize the ERG for disease classification or early detection in psychiatric disorders [14, 15] provides another methodological approach to identifying functional biomarkers in neurological disorders [14].

The application of synthetic signal generation to the ERG in supporting improved classification of these groups may provide the potential to expand the clinical applications of the ERG in psychiatric disorders [15, 61]. The scope of practice for ERG analysis now encompasses therapeutics in major depression [62], ADHD [11, 63], schizophrenia [4] and Parkinson’s Disease [6, 7]. Synthetic reference signals have demonstrated clinical benefits and enhanced medical operational efficiency by offering a feasible alternative to natural signals [20, 22]. Synthesizing additional signals further facilitates dataset expansion within specialized domains, enabling training resource intensive networks such as transformers for improving healthcare [64].

One limitation of this population sample is that the responses of individuals under 5 years of age, when a clinical biomarker would be most advantageous, have yet to be performed, though technically possible [2, 3, 65]. Furthermore, a direct comparison of the time domain parameters between the real and synthetic ERGs in a wider range of waveforms would be the next step to validate further the characteristics of the synthetic waveforms fully. Despite these current limitations, the incorporation of synthetic signals generated by the proposed CGAN model offers a promising solution to address existing challenges in the domain of AI applied to the ERG. Expanding the number of samples collected from subjects and generating synthetic waveforms provides a new opportunity to expand AI modeling of the ERG in rare or heterogeneous populations [66] where large datasets are required. The augmentation with synthetic signals will provide the opportunity to train heavy models such as Transformers to support the early detection of retinal disorders. Furthermore, the non-personal nature of synthetic signals permits their open-source publication, making them suitable for sharing without violating patient privacy concerns.

## Data Availability

Raw data are available from the public repository https://doi.org/10.25451/flinders.27116719.v1 and the synthetic dataset is available at IEEE DataPort [1].
